# Expanding the phenotypic spectrum of non-alcoholic fatty liver disease and hypertriglyceridemia

**DOI:** 10.3389/fnut.2022.967899

**Published:** 2022-09-15

**Authors:** Marica Meroni, Miriam Longo, Erika Paolini, Giada Tria, Michela Ripolone, Laura Napoli, Maurizio Moggio, Anna Ludovica Fracanzani, Paola Dongiovanni

**Affiliations:** ^1^General Medicine and Metabolic Diseases, Fondazione IRCCS Cà Granda Ospedale Maggiore Policlinico, Milan, Italy; ^2^Department of Clinical Sciences and Community Health, Università degli Studi di Milano, Milan, Italy; ^3^Department of Pharmacological and Biomolecular Sciences, Università degli Studi di Milano, Milan, Italy; ^4^Neuromuscular and Rare Diseases Unit, Fondazione IRCCS Ca’ Granda Ospedale Maggiore Policlinico, Milan, Italy; ^5^Department of Pathophysiology and Transplantation, Università degli Studi di Milano, Fondazione IRCCS Cà Granda Ospedale Maggiore Policlinico, Milan, Italy

**Keywords:** hypertriglyceridemia, NAFLD, whole-exome sequencing, transmission electron microscopy, mitochondrial dysfunction

## Abstract

**Background and aims:**

Hypertriglyceridemia is a common feature of metabolic syndrome (MetS), as well as of non-alcoholic fatty liver disease (NAFLD), which is considered the hepatic manifestation of MetS. Fat accumulation in hepatocytes may alter mitochondrial homeostasis predisposing to advanced liver disease. Here, we report a case of a 40-year-old woman with early aggressive NAFLD due to severe hypertriglyceridemia that ensued from a combination of genetic variants and additional metabolic risk factors.

**Methods:**

Genetic screening was performed by using whole-exome sequencing (WES), and mitochondrial structures were evaluated by TEM.

**Results:**

At presentation, the patient is reported to have hepatomegaly, hypertriglyceridemia, and raised transaminases. Genetic analysis revealed that the patient beard heritable alterations in genes implicated in lipid handling, among which *APOB*, *APOE*, *CETP*, and *HSPG2*, accompanied by missense mutations in genes involved in mitochondrial function, i.e., *AK2, ALG6, ASPA, NDUFAF1, POLG*, and *TMEM70*. Abdominal ultrasound (US) and transient elastography were suggestive of severe hepatic steatosis and fibrosis. A liver biopsy confirmed the diagnosis of non-alcoholic steatohepatitis (NASH)-related fibrosis. Thus, to better outline whether mutations involved in lipid remodeling and mitochondrial function may also affect organelles’ morphology, we exploited TEM. Along with multifaceted abnormalities of mitochondrial architecture that have been already observed in patients with NAFLD, astonishing ultrastructural defects, such as mitochondrial vacuolization, sub-compartmentalization, and *onion-like* mitochondria, were identified.

**Conclusion:**

The anomalies reported may expand the phenotypic spectrum of mitochondrial abnormalities observed in patients with NAFLD, which may contribute to the switching toward a progressive disease.

## Introduction

Hypertriglyceridemia is a hallmark of metabolic syndrome (MetS) along with obesity, insulin resistance (IR), hypertension, high blood sugar, and abnormal cholesterol levels (1). Plasma triglycerides (TGs) are gathered in diet-derived chylomicrons and liver-synthetized very low-density lipoprotein (VLDL) particles (2). Atherogenic dyslipidemia, characterized by elevated TGs (>150 mg/dL) and low high-density lipoprotein (HDL) concentrations, is a feature of non-alcoholic fatty liver disease (NAFLD), which represents the hepatic manifestation of MetS (1, 3). NAFLD pathogenesis is convoluted, and it is severely influenced by environmental and inherited jeopardizing factors (4). An unbalanced diet, especially enriched in saturated fatty acids, and a sedentary lifestyle enormously precipitate hepatic fat accumulation, as well as extrahepatic comorbidities (5). Nutrigenomic studies highlighted the impact of human genetic variations on nutrient utilization/metabolism, food tolerances, and micronutrient requirement, thus suggesting that the individual’s genetic background may shape the risk of developing NAFLD and dyslipidemia by consuming different types of foods (4). Furthermore, recent data demonstrated that serum/hepatic lipidome is extensively influenced by genetic modifiers and correlates with mitochondrial dysfunction and disease progression in preclinical models of NAFLD and in patients (6, 7). Indeed, a possible pathogenic mechanism linking hypertriglyceridemia and severe liver disease relies on defects in mitochondrial homeostasis (8). Mitochondria bear the ability to adapt their activity and biomass, in response to highly energetic substrates due to a hypercaloric diet and adipose tissue IR, which prompt enhanced flux of free fatty acids (FFAs) to the liver. This phenomenon is referred to as “mitochondrial flexibility” (9). However, this flexibility is lost in the transition toward more severe liver damage, and the mitochondrial efforts to sustain β-oxidation may not be bioenergetically efficient to compensate for energy demand (10, 11), thus triggering, in turn, exasperated reactive oxygen species (ROS) production, DNA damage, and phospholipid lipoperoxidation. Hence, these events may favor organelle membrane disruption and lipid droplet (LD) expansion (12, 13).

Here, we present a case of a 40-year-old woman with early aggressive NAFLD development due to severe hypertriglyceridemia, which resulted from a combination of genetic variants and additional metabolic risk factors.

## Case presentation

The female patient is noted to have dyslipidemia with raised TGs until 1,087 mg/dL. She was recommended to our outpatient service by her general practitioner for dyspepsia due to an 8-mm esophageal granular cell benign tumor, accompanied by an elevation of alanine aminotransferase (ALT) to 69 U/L, aspartate aminotransferase (AST) to 154 U/L, gamma-glutamyl transferase (GGT) to 624 U/L, and alkaline phosphatase (ALP) to 136 U/L, on routine testing. She reported a familial history of gastric cancer, and she took medications for gastric protection (40 mg/die).

Dietary history was noteworthy for excess carbohydrates and saturated and trans fats, together with sugar beverages (up to seven liters *per* week), chocolate, and candies. She denied consuming wine and liquor. She referred to having been obese in the past [weight 90 kg, body mass index (BMI) of 29.4 kg/m^2^], and scant physical activity. Abdominal adiposity is noted. The patient was a smoker with an estimated 10 cigarettes per day smoking history, and she did not follow the nutritional advice from the clinicians.

At presentation, the physical examination revealed blood pressure of 130/80 mmHg, 85 beats per min, weight of 71 kg, BMI of 23.2 kg/m^2^, and an abdominal circumference of 130 cm. The lipid panel (most recent) showed total cholesterol of 308 mg/dL, HDL-c of 42 mg/dL, and LDL-c of 270 mg/dL. Neither ascites nor jaundice were detectable. Abdominal ultrasound (US) indicated severe hepatomegaly, hyperechogenic liver without focal abnormalities, associated with steatosis (S3), normal portal vein caliber, and minor splenomegaly (130 mm). The liver stiffness, measured by transient elastography (FibroScan), was 10.7 kPa (F3), and the CAP was 313 dB/m (S3).

Viral serological markers (hepatotropic viruses, Epstein-Barr virus, cytomegalovirus, and HIV), toxoplasma, thyroid function tests, creatine phosphokinase, glucose, ceruloplasmin, serum copper, urine routine tests, blood ammonia, creatinine, uric acid, autoimmune and celiac serology, and immunoglobulin levels were unremarkable. Coagulation studies and albumin were within normal ranges.

Therefore, the patient underwent to US-guided liver biopsy to confirm the diagnosis of non-alcoholic steatohepatitis (NASH)-related fibrosis. The liver specimen was evaluated by an expert pathologist unaware of the patient’s disease *status* and genetic background. The histological assessment confirmed the non-invasive conclusions, evidencing marked panlobular micro-macrovesicular steatosis on hematoxylin and eosin-stained sections, which was diffused in 70–80% of hepatic parenchyma (S3). Steatosis was also related to lobular inflammation (grade 1), scattered patchy necrosis, rare Mallory-Denk bodies, mild marginal ductular reaction, and focal hepatocellular ballooning (grade 1), indicative of the presence of NASH with a NAFLD Activity Score (NAS) equal to 5. Iron depots were absent. Incomplete septa were moderately diffused into the hepatic tissue, related to centrilobular and perisinusoidal fibers, and suggestive of advanced fibrosis (F3). All biochemical and histopathological parameters of the patient are listed in Table 1.

**TABLE 1:** Clinical and laboratory findings of a 40-year-old woman with non-alcoholic fatty liver disease.

Clinical parameter	Results	Normal range
Presenting age	40 years	–
Height (cm)	175 cm	–
Weight (kg)	71 kg	–
Abdominal circumference (cm)	130 cm	[40–80]
BMI (kg/m^2^)	23.1 kg/m^2^	[18.5–24.9]
Heart rate (beats per minute)	85 bpm	[60–100]
Blood pressure (mmHg)	130/80 mmHg	120/80 mmHg
Active smoke	10 cigarettes/day	Absence
Hepatomegaly	Severe	Absence
Splenomegaly	Minor	Absence
Albumin (g/dL)	4.4 g/dL	[3.4–5.4]
ALT (U/L)	69 U/L	[6–41]
AST (U/L)	154 U/L	[10–33]
GGT (U/L)	624 U/L	[5–36]
ALP (U/L)	136 U/L	[35–104]
Direct bilirubin (mg/dL)	0.48 mg/dL	[0.0–0.30]
Total bilirubin (mg/dL)	1.17 mg/dL	[0.12–1.10]
Lactate (mmol/L)	3.76 mmol/L	[0.5–2.20]
LDH (U/L)	496 U/L	[135–214]
Triglycerides (mg/dL)	1087 mg/dL	<150 mg/dL
Total cholesterol (mg/dL)	308 mg/dL	<190 mg/dL
HDL-c (mg/dL)	42 mg/dL	>45 mg/dL
LDL-c (mg/dL)	270 mg/dL	<145 mg/dL
Lipoprotein (a) (nmol/L)	14.17 (nmol/L)	<75 nmol/L
Glucose (mg/dL)	92 mg/dL	[70–110]
Urea (mg/dL)	14 mg/dL	[15–50]
Uric Acid (mg/dL)	6.9 mg/dL	[2.4–5.7]
Creatinine (mg/dL)	0.7 mg/dL	[0.55–0.96]
Creatine phosphokinase (U/L)	125 U/L	[60–190]
Creatine Kinase (U/L)	68 U/L	[22–198]
Thyroid stimulating hormone (TSH) (mIU/L)	1.94 mIU/L	[0.28–4.30]
Ferritin (μg/L)	129 μg/L	[15–150]
Ceruloplasmin (g/L)	0.226 g/L	[0.2–0.60]
Serum copper (μg/L)	1264 μg/L	[600–1600]
Serum iron (μg/dL)	153 μg/dL	[60–170]
Folic acid (μg/L)	16.9 μg/L	[4.6–18.7]
Vitamin B12 (ng/L)	603 ng/L	[191–663]
Activated partial thromboplastin time (s)	32.5 s	[21–35]
Prothrombin time (s)	11.2 s	[11–13.5]
Fibrinogen (mg/dL)	213 mg/dL	[165–350]
Hemoglobin (g/dL)	13.4 g/dL	[12–16]
Red blood cell count (per liter)	4.26*10^12/L	[4.1–5.1]
White blood cell count (per liter)	4.83*10^9/L	[4.8–10.8]
Lymphocytes (%)	35%	[20–40]
Neutrophils (%)	56.3%	[40–60]
Platelets count (per liter)	119*10^9/L	[130–400]
Steatosis grade (US)	3	Absence
CAP (dB/m)	313 dB/m	[150–248]
Liver stiffness (kPa)	10.7 kPa	[2.0–8.5]
Histological steatosis	3	Absence
Histological lobular inflammation	1	Absence
Histological ballooning	1	Absence
NAFLD activity score (NAS)	5	Absence
Histological fibrosis	3	Absence

Since plasma TG concentrations are heavily influenced by heritable variants, we decided to perform a whole-exome sequencing (WES). Genomic DNA (gDNA) was extracted from peripheral blood mononuclear cells (PBMC), according to the manufacturer’s standard procedure, using the QIAamp DNA Blood Mini Kit (QIAGEN, Germany). The Fluorometer Qubit 4.0 (Thermo Fisher Scientific, United States) was used for DNA quantification. Next-generation sequencing (NGS) was performed using the Illumina NextSeq 550 System. DNA was fragmented and the target sequences were enriched by PCR methods (SureSelect Technology). All single-nucleotide variants were filtered via multiple databases and variants frequency was evaluated by dbSNP, using European descent as the reference population. The pathogenicity of gene variations was evaluated through *in silico* prediction programs (CADD and Polyphen-2). All variations that emerged were confirmed by Sanger sequencing (3130 Genetic Analyzer, Thermo Fisher Scientific), according to the manufacturer’s protocol.

The patient harbored a cluster of multiple inherited variations in canonical genes regulating TG metabolism, among which those in *ABCA1, ABCG8, ACADM, APOB, APOC3, APOE, CETP, CPT1A, HSPG2, LARS1, LIPI, MTTP, PCSK9*, and *PNPLA3.* The complete list of the above-mentioned genetic variants is represented in Table 2. Taken together, these mutations may constitute the determinants of higher plasma TGs observed in this patient.

**TABLE 2:** Possibly pathogenic rare and common variants involved in hypertriglyceridemia development, identified in the 40-year-old woman with NAFLD.

Gene	Transcript	rsID	cDNA	Protein	MAF	Variant ontology	Genotype	CADD	Polyphen-2
*ABCA1*	NM_005502.4	rs1800978	c.-18G > C	–	G = 0.08	5′UTR	C/G	15.35	
*ABCG8*	NM_022437.3	rs4148211	c.161A > G	p.Tyr54Cys	G = 0.38	Exonic, Missense	A/G	23.50	0.436
*ABCG8*	NM_022437.3	rs6544718	c.1895T > C	p.Val632Ala	T = 0.21	Exonic, Missense	T/C	11.90	0.0
*ACADM*	NM_000016.5	rs77931234	c.985A > G	p.Lys329Glu	G = 0.007	Exonic, Missense	A/G	22.90	0.071
*APOB*	NM_000384.2	rs1042034	c.13013G > A	p.Ser4338Asn	C = 0.21	Exonic, Missense	C/T	1.89	0.0
*APOB*	NM_000384.2	rs676210	c.8216C > T	p.Pro2739Leu	A = 0.21	Exonic, Missense	G/A	27.40	1.0
*APOB*	NM_000384.2	rs679899	c.1853C > T	p.Ala618Val	A = 0.47	Exonic, Missense	G/A	24.80	1.0
*APOB*	NM_000384.2	rs1367117	c.293C > T	p.Thr98Ile	A = 0.29	Exonic, Missense	A/A	22.0	0.125
*APOC3*	NM_000040.3	rs2070666	c.179 + 62T > A	-	A = 0.09	Intronic	A/A	1.16	
*APOE*	NM_000041.4	rs7412	c.526C > T	p.Arg176Cys	T = 0.08	Exonic, Missense	C/T	25.00	1.0
*CETP*	NM_000078.3	rs752298084	c.1046C > A	p.Ser349Tyr	A = 0.0001	Exonic, Missense	C/A	16.74	0.99
*CPT1A*	NM_001876.4	rs61887062	c.693 + 37T > C	–	G = 0.00	Intronic	G/G	6.83	
*HSPG2*	NM_001291860.2	rs62642528	c.3059C > T	p.Pro1020Leu	A = 0.01	Exonic, Missense	G/A	22.60	0.99
*HSPG2*	NM_001291860.2	rs766963773	c.2039G > A	p.Arg680Hys	T = 0.0001	Exonic, Missense	C/T	25.90	0.99
*LARS1*	NM_020117.11	rs112912805	c.2578G > A	p.Ala860Thr	T = 0.004	Exonic, Missense	C/T	22.10	0.008
*LIPI*	NM_001302998.2	rs2822432	c.1291G > A	p.Glu431Lys	T = 0.34	Exonic, Missense	C/T	23.60	0.57
*MTTP*	NM_000253.3	rs3792681	c.394-32T > C	–	C = 0.03	Intronic	T/C	16.20	
*MTTP*	NM_000253.3	rs11734413	c.502-42C > T	–	T = 0.09	Intronic	C/T	5.90	
*PCSK9*	NM_174936.3	rs11800231	c.524-11G > A	–	A = 0.05	Intronic	G/A	0.33	
*PCSK9*	NM_174936.3	rs11800243	c.657 + 9G > A	–	A = 0.05	Intronic	G/A	5.40	
*PCSK9*	NM_174936.3	rs11800265	c.658-36G > A	–	A = 0.05	Intronic	G/A	7.40	
*PCSK9*	NM_174936.3	rs505151	c.2009G > A	p.Gly670Glu	G = 0.04	Exonic, Missense	G/A	3.67	0.0
*PNPLA3*	NM_025225.3	rs738409	c.444C > G	p.Ile148Met	G = 0.21	Exonic, Missense	C/G	15.73	0.99

Since mitochondria constitute the cell’s powerhouse, thus orchestrating the maladaptive response to hypertriglyceridemia, we then focused our attention on those candidate gene *loci*, which impact on mitochondrial biology. We highlighted that the patient carried a broad number of genetic variants, influencing mitochondrial homeostasis, including those in *AK2*, *ALG6*, *ALG8*, *ARSB*, *ASPA*, *CPN1*, *DHTKD1*, *ETFA*, *FARS2*, *GAA*, *GFM1*, *HSD17B4*, *NDUFAF1*, *NDUFS3*, *NNT, POLG*, and *TMEM70*. The complete list of these mutations is represented in Table 3.

**TABLE 3:** Possibly pathogenic rare and common variants involved in mitochondrial biological processes, identified in the 40-year-old woman with NAFLD.

Gene	Transcript	rsID	cDNA	Protein	MAF	Variant ontology	Genotype	CADD	Polyphen-2
*AK2*	NM_001319140.2	rs113711467	c.458A > T	p.Tyr153Phe	A = 0.10	Exonic, Missense	T/A	24.90	0.99
*ALG6*	NM_013339.4	rs35383149	c.391T > C	p.Tyr131His	C = 0.04	Exonic, Missense	T/C	23.30	0.014
*ALG6*	NM_013339.4	rs41285372	c.1357C > G	p.Leu453Val	G = 0.019	Exonic, Missense	C/G	23.50	0.98
*ALG8*	NM_001007027.3	rs665278	c.665A > G	p.Asn222Ser	C = 0.23	Exonic, Missense	C/C	15.94	0.0
*ARSB*	NM_000046.5	rs25414	c.1151G > A	p.Ser384Asn	T = 0.06	Exonic, Missense	C/T	19.30	0.001
*ASPA*	NM_00049.3	rs28940574	c.914C > A	p.Ala305Glu	A = 0.0005	Exonic, Missense	C/A	25.30	1.0
*CPN1*	NM_00138.3	rs61751507	c.533G > A	p.Gly178Asp	T = 0.05	Exonic, Missense	C/T	22.70	0.78
*DHTKD1*	NM_018706.7	rs776400922	c.1986G > T	p.Glu662Asp	A = 0.00000	Exonic, Missense	G/T	T = ?	0.55
*ETFA*	NM_000126.4	rs141200145	c.826A > C	p.Ile276Leu	G = 0.002	Exonic, Missense	T/G	22.80	0.07
*FARS2*	NM_001374877.1	rs11243011	c.839A > G	p.Asn280Ser	G = 0.18	Exonic, Missense	G/G	17.51	0.01
*GAA*	NM_000152.3	rs1800309	c.2065G > A	p.Glu689Lys	A = 0.04	Exonic, Missense	G/A	18.60	0.028
*GFM1*	NM_001308164.2	rs34297061	c.476A > G	p.Asn159Ser	G = 0.00015	Exonic, Missense	A/G	14.43	0.006
*GFM1*	NM_001308164.2	rs62288347	c.2047G > A	p.Val683Ile	A = 0.017	Exonic, Missense	G/A	23.70	0.49
*HSD17B4*	NM_001199291.3	rs25640	c.392G > A	p.Arg131His	A = 0.46	Exonic, Missense	G/A	32.00	0.99
*NDUFAF1*	NM_016013.4	rs12900702	c.941C > G	p.Ala314Gly	C = 0.18	Exonic, Missense	G/C	26.20	0.95
*NDUFS3*	NM_004551.3	rs148331180	c.475G > C	p.Val159Leu	C = 0.004	Exonic, Missense	G/C	19.90	0.004
*NNT*	NM_012343.4	rs78818665	c.2977A > G	p.Ile993Val	G = 0.006	Exonic, Missense	A/G	25.80	0.95
*POLG*	NM_001126131.2	rs2307441	c.3428A > G	p.Glu1143Gly	C = 0.04	Exonic, Missense	C/C	22.80	0.98
*TMEM70*	NM_001040613.2	rs8075	c.100G > C	p.Ala34Pro	C = 0.15	Exonic, Missense	C/C	17.20	0.92

Hence, given the peculiar heritable asset of this patient, which may affect mitochondrial function/structure, we next decided to better outline the impact of these mutations on mitochondrial morphology by using TEM. Hepatic biopsy was fixed in 2.5% glutaraldehyde in cacodylate buffer pH 7.4, overnight. Afterward, it was postfixed in 2% osmium tetroxide (OsO_4_) for 1 h. Finally, the liver specimen was dehydrated with increasing ethanol series, embedded in an Epon resin, and polymerized in an oven at 62°C for 48 h. Ultrathin (70–90 nm) sections were collected on nickel grids and observed with a Zeiss EM109 (14).

**FIGURE 1:**
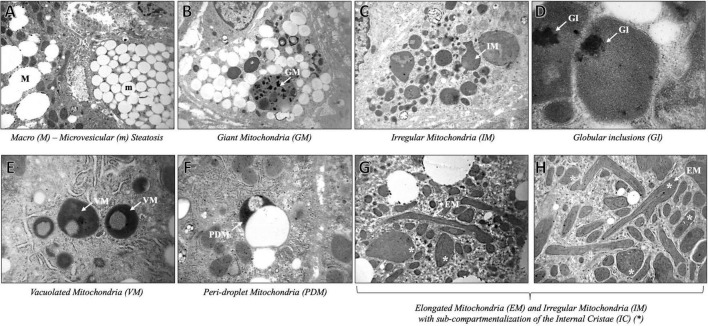
Representative TEM images of mitochondrial ultrastructure aberrancies in a 40-year-old patient with non-alcoholic fatty liver disease, obtained by ultrathin 70-nm sections of hepatic biopsy. White arrows indicate the anomalies identified as follows: Macro (M) – microvesicular (m) steatosis **(A)** (3,000× magnification), Giant Mitochondria (GM) **(B)** (4,400×); Irregular Mitochondria (IM) **(C)** (3,000×); Globular Inclusions (GI) **(D)** (20,000×); Vacuolated Mitochondria (VM) **(E)** (7,000×); Peri-droplet Mitochondria (PDM) **(F)** (7,000×); Elongated Mitochondria (EM), and IM with high-packed cristae and dense matrix, partitioned by the abnormal Internal Cristae (IC) (*) arrangement **(G)** (3,000X), **(H)** (4,440×).

**FIGURE 2:**
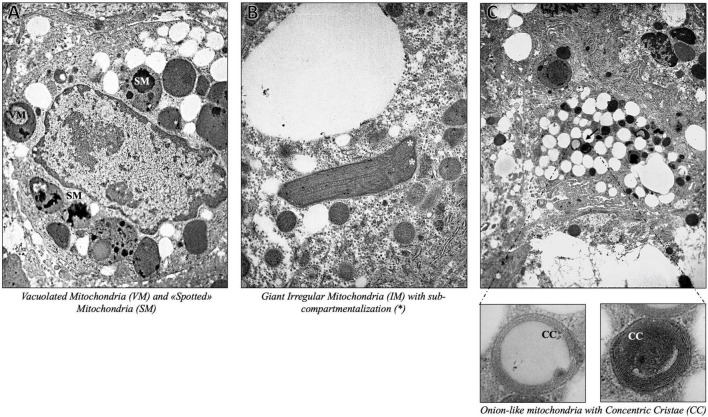
Representative TEM images of onion-like giant mitochondria, defined by swirling layers of cristae membranes, obtained by ultrathin 70-nm sections of hepatic biopsy. Black arrows indicate the anomalies identified as follows: Vacuolated Mitochondria (VM) and “Spotted” Mitochondria (SM) **(A)** (7,000×), Giant Mitochondria with sub-compartmentalization (*) **(B)** (7,000×), Onion-like Mitochondria with Concentric Cristae (CC) **(C)** (4,400× magnification with inserts at 30,000×).

As expected, we found severe fat depots that involve the majority of the cytoplasm of hepatocytes, resulting in micro- and macrovesicular steatosis and abundant glycogen storages. The hepatic parenchyma was even featured by multifaceted abnormalities of mitochondrial architecture already observed in aggressive NAFLD, such as megamitochondria, elongated mitochondria, and irregular mitochondrial morphology with globular inclusions (Figure 1). Intriguingly, the alterations of the mitochondrial matrix and atypical arrangement of inner mitochondrial cristae have been detected in this patient and were never described in both hypertriglyceridemia and NAFLD disorders. Spherical vacuoles, a sign of irreversible cell injury reported in an experimental model of amyotrophic lateral sclerosis (ALS) (15), often accompanied by highly electron-dense bodies within giant mitochondria (coined as “*spotted mitochondria*”) and close to LDs (peri-droplet mitochondria) were recurrently found in hepatocytes. Likewise, o*nion-like* mitochondria, defined by concentric layers of cristae membranes, and giant mitochondria characterized by both extremely thick matrix and aberrancies of the inner cristae organization, which seem to induce a mitochondrial sub-compartmentalization, were frequently observed (Figure 2). Therefore, aberrant mitochondria were more recurrent compared to their normal-sized counterparts. Mitochondrial morphology is intrinsically linked to the preservation of their correct biology in human tissues and in particular, cristae membranes are dynamic, reshaping themselves through membrane fission and fusion events. The liver is physiologically enriched in mitochondria, thus classically sustaining oxidative phosphorylation and ATP synthesis in response to energy demand. Here, we reported abnormal features not previously depicted in human hepatic tissues, thus expanding the spectrum of mitochondrial aberrancies in NAFLD. Nonetheless, cristae membranes may be also affected by the lipid microenvironment or by genetic mutations in the *OPA1* gene, encoding the Mitochondrial Dynamin Like GTPase responsible for the regulation of cristae dynamism (16) or by mitochondrial DNA depletion (17). However, any mutations in the *OPA1* gene have been found in this patient, and the genetically determined number of mitochondrial DNA copies evaluated through the D-loop (data not shown) was in the normal range. According to these abnormalities, we evidenced high levels of blood lactate and lactate dehydrogenase (LDH) reaching 3.76 mmol/L and 496 U/mL, respectively. The latter may be suggestive of impaired oxygen consumption and elevated anaerobic metabolism.

## Discussion

Severe hypertriglyceridemia is intertwined with obesity, MetS, and diabetes. Nonetheless, even heritable variants in genes regulating TG-lipoproteins’ synthesis, release, or clearance, may exert a key role in its development. Here, we reported a case of a 40-year-old woman with early-onset NAFLD due to hypertriglyceridemia which resulted from a combination of inherited mutations and additional metabolic risk factors. The genetic screening demonstrated that this patient carried a broad series of heritable alterations that concomitantly occur, whereby possibly explaining the biochemical phenotype. Indeed, it has been reported that severe inherited hypertriglyceridemia is more likely dependent on a polygenic etiology. Specifically, an increased frequency of homozygous/heterozygous variants is a hallmark of patients with hypertriglyceridemia (18). This phenomenon is known as “variable” or “incomplete” penetrance, which differs from the “complete” penetrance that characterizes the majority of cases of familial hypercholesterolemia (19). Thus, the co-occurrence of environmental jeopardizing factors may be deterministic, amplifying the risk to ascertain the clinical phenotype.

Since fat accumulation in hepatocytes may lead to mitochondrial dysfunction in terms of uncoupling oxidative phosphorylation and mitochondrial permeability transition, we focused our attention on candidate gene *loci*, which impact on mitochondrial biology and on the adaptation of mitochondria to the context of severe TG overload, since they are highly dynamic organelles bearing essential metabolic and regulatory activities (16, 20). A plethora of genetic variants, that may influence mitochondrial function, and multifaceted ultrastructural anomalies have been observed. As a result of fat accumulation into the hepatocytes, mitochondria are found to be in close contact with LDs (referred to as “peri-droplet mitochondria”), possibly supporting either ATP-dependent triacylglycerol synthesis (20) or sustaining fat disposal (21). Furthermore, TEM microscopy allowed us to visualize the presence of giant mitochondria in the liver, displaying a distinctly modified internal structural appearance, paralleled by intense vacuolization and/or large dark inclusions. Giant mitochondria with atypical membrane distribution and highly packed appearance were also noteworthy signs, as the cristae seem to the round-shape mitochondrial internal matrix, resulting in multiple-compartment mitochondria. Mitochondrial gigantism is a peculiar phenomenon observed in hepatic biopsies of patients with NAFLD, as a consequence of exaggerated fat accumulation (22). Likewise, mitochondrial enlargement with crystalline inclusions is considered a warning signal of degeneration, paralleled by mitochondria autophagic engulfment and degradation (23). Alongside, unusual ultrastructural defects, resembling mitochondria with *onion-like* swirling cores were firstly pinpointed in the context of NAFLD. Proper architecture and arrangement of the inner mitochondrial membranes are essential for the effectiveness of respiration and apoptosis’ quality control (24). In detail, cristae are highly convoluted and dynamic structures, in terms of number and dimension, modifying themselves according to the energetic state of the cells. The establishment of mitochondrial membrane architecture is strictly dependent on the evolutionarily conserved machinery that is involved in mitochondrial cristae biogenesis, encompassing the dimerization of the mitochondrial ATP synthase, the shaping of the mitochondrial contact site and cristae organizing system (MICOS), the remodeling of the inner membrane by OPA1, and the modulation of the mitochondrial lipid composition (25). A growing body of evidence pointed out the critical role of mitochondrial ultrastructural deteriorations in the pathogenesis of progressive NAFLD, raising the possibility that it may be considered a *mitochondrial disorder* (6, 8, 26). However, the abnormal features reported here have not been previously depicted in human hepatic tissues, thus expanding the spectrum of mitochondrial aberrancies in NAFLD and although we did not observe any possible causal mutations in genes regulating mitochondrial inner membranes’ assembly, we cannot rule out the presence of additional mitochondrial DNA defects in terms of both hepatic content and presence of inherited/acquired mutations.

## Concluding remarks

In conclusion, we demonstrated that inherited variants, which impact on TG remodeling and clearance, may be causative of severe hypertriglyceridemia in patients with NASH. Furthermore, lipid accumulation in hepatocytes together with mutations in genes implicated in mitochondrial function, which in turn may affect the organelles’ structure, might force their aggressive derangements, thus fostering the early-onset and the severity of liver dysfunction.

## Data availability statement

The data presented in this study are deposited in the National Library of Medicine repository [Sequence Read Archive (SRA)], accession number: SRR21201235 (https://www.ncbi.nlm.nih.gov/). BioProject ID: PRJNA873353, BioSample Accession: SAMM30496948.

## Ethics statement

The studies involving human participants were reviewed and approved by Fondazione IRCCS Cà Granda Ospedale Maggiore Policlinico. The patients/participants provided their written informed consent to participate in this study.

## Author contributions

MMe and ML participated in the design of this study, performed experiments and the analysis, and drafted the manuscript. EP and GT participated in the experiments, analyzed, and critically reviewed the manuscript. MR, LN, and MMo performed the TEM analysis. AF recruited patients for the study and contributed to the critical review of the manuscript. PD participated in the conceptual design of the study, interpreted data, and contributed to the critical review of the manuscript. All authors contributed to the article and approved the submitted version.

## Abbreviations

US: abdominal ultrasound
ALT: alanine aminotransferase
ALP: alkaline phosphataseALP
AST: aspartate aminotransferase
BMI: body max index
GGT: gamma-glutamyl transferase
HDL: high-density lipoprotein
IR: insulin resistance
LDH: lactate dehydrogenase
LD: lipid droplet
MetS: metabolic syndrome
NGS: next-generation sequencing
NAFLD: non-alcoholic fatty liver disease
NASH: non-alcoholic steatohepatitis
PBMC: peripheral blood mononuclear cells
TEM: transmission electron microscopy
TGs: triglycerides
VLDL: very low-density lipoprotein.
